# Iatrogenic Rhinolith: A Case Report and Review of Literature

**DOI:** 10.5811/cpcem.1395

**Published:** 2023-05-26

**Authors:** Daniel Mitchell, Quinn Self, Carolyn Orgain

**Affiliations:** * The University of Vermont - Larner College of Medicine, Burlington, Vermont; † The University of Vermont Health Network, Department of Otolaryngology Surgery, Burlington, Vermont

**Keywords:** Rhinolith, nasal mass, epistaxis, nasal drainage, case report

## Abstract

**Introduction:**

Unilateral nasal obstruction is a common complaint with a broad differential diagnosis that includes anatomic asymmetry, unilateral infective or inflammatory conditions, and benign and malignant sinonasal masses. A rhinolith is an uncommon foreign body in the nose, which serves as a nidus for calcium salt deposition. The foreign body can be endogenous or exogenous in origin and may remain asymptomatic for many years before incidental discovery. When left untreated, stones may cause unilateral nasal obstruction, rhinorrhea, nasal discharge, epistaxis or, in rare cases, progressive destruction leading to septal/palatal perforation or oro-antral fistula. Surgical removal is an effective intervention with limited complications reported.

**Case Report:**

This article describes a 34-year-old male who presented to the emergency department (ED) with unilateral obstructing nasal mass and epistaxis, which was found to be an iatrogenic rhinolith. Successful surgical removal was performed.

**Conclusion:**

Epistaxis and nasal obstruction are common presentations to the ED. Rhinolith is an uncommon clinical etiology that if left undiagnosed may lead to progressive destructive disease; it should be included in the differential for any unilateral nasal symptoms of unclear origin. Appropriate work-up for any suspected rhinolith includes computed tomography, as biopsy is risky given the broad differential of unilateral nasal mass. When identified, surgical removal has a high success rate with limited complications reported.

## INTRODUCTION

Nasal obstruction and epistaxis are common complaints in acute care medicine. Obstruction may be acute or chronic, bilateral or unilateral, and may be anatomical or inflammatory in origin.^1^ Common causes of unilateral nasal obstruction include anatomic asymmetries, allergic or infectious sinusitis, and benign or malignant neoplastic processes.^2^ It is often difficult to differentiate the etiology of nasal masses on physical examination alone. Any unilateral nasal mass should undergo imaging and referral to otolaryngology for biopsy.^2^–^4^

A rhinolith is a rare, often overlooked etiology for unilateral nasal obstruction, epistaxis, and nasal drainage that presents similarly to neoplastic nasal masses on physical examination.^5^ A rhinolith forms when an endogenous or exogenous foreign body serves as a nidus for calcium deposition over many years.^6^ Computed tomography (CT) is useful for diagnosis and assessment of size and local mass effect, as well as for sur gical approach decision-making.^7^,^8^ Complication following stone removal is rare and typically limited to bleeding, local infection, or ipsilateral sinusitis.^6^

We present a case of iatrogenic rhinolith in a neurocognitively intact adult male likely arising from retained packing after an intranasal operation 2.5 years prior in Afghanistan, and we discuss clinical, pathological, and radiological features.

## CASE REPORT

**Image 1:**
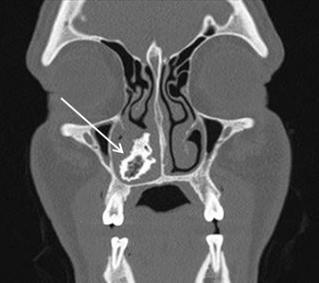
Coronal computed tomography demonstrating calcified mass indicated by white arrow in the inferior meatus.

A 34-year-old male who had recently immigrated from Afghanistan presented to the emergency department (ED) with several years of right-sided nasal obstruction and several days of intermittent self-limiting right-sided epistaxis. Past medical and surgical history was significant for an intranasal procedure 2.5 years prior while still in Afghanistan. Initial evaluation by the emergency physician revealed a whitish-gray, rock-hard mass in the floor of the right nasal passage, which was biopsied for culture. A CT was also obtained. Biopsy revealed aggregates of filamentous, Gram-positive rods with short, modified acid-fast microorganisms. Computed tomography revealed a 3.5-centimeter calcified mass inferior to the right turbinate (Image 1).

**Image 2:**
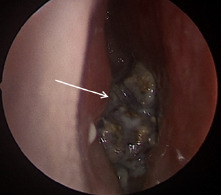
Anterior rhinoscopy demonstrating calcified mass indicated by white arrow.

**Image 3:**
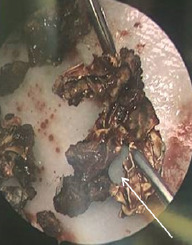
Specimen with calcified exterior and synthetic rubbery core indicated by white arrow.

Outpatient referral and subsequent surgical removal was recommended by otolaryngology. Due to physical exam and imaging characteristics, antibiotics were not recommended. The patient ultimately underwent uncomplicated surgical removal. Intraoperatively a large, calcified nasal mass was encountered between the septum and inferior turbinate (Image 2). This was adherent to the surrounding nasal mucosa. With removal, friable mucosa was exposed, and mucosal bleeding was encountered that was controlled with topical vasoconstrictors. The foreign body was removed piecemeal and demonstrated a calcified exterior and a synthetic rubbery center (Image 3).

He recovered without complication.

*CPC-EM Capsule*

What do we already know about this clinical entity?

*Rhinoliths are benign intranasal calcified foreign bodies that often present with nonspecific symptoms such as epistaxis or nasal obstruction with an indolent course*.

What makes this presentation of disease reportable?

*A neurocognitively intact adult presented with epistaxis and intranasal mass. Removal suggested retained surgical material from history of nasal surgery*

What is the major learning point?

*There is a broad differential for intranasal masses including benign and malignant etiologies. Imaging and subspecialty consultation are often indicated*.

How might this improve emergency medicine practice?

*Rhinoliths are uncommon, difficult to visualize and are nonspecific in presentation. Definitive management is removal. They should remain on the differential for nasal complaints*.

## DISCUSSION

A rhinolith is a calcification in the nasal passage that originates when a foreign body serves as a nidus for inorganic salt deposition.^9^ Rhinoliths are uncommon and may be overlooked and underdiagnosed, as the estimated prevalence is approximately 1 in 10,000 patients.^10^ Nasal foreign body is a common finding in children and neurocognitively impaired adults when a bead or similar object is manually placed into the nose.^5^ Rhinoliths, however, are seen across all age groups and mental states. Given the time required for stones to form, they are most often reported in adults between the third and sixth decades of life.^11^,^12^

Rhinoliths may be found incidentally on anterior rhinoscopy, nasal endoscopy, or imaging studies when asymptomatic.^6^ Symptoms of occlusive stones may include unilateral nasal obstruction, purulent rhinorrhea, ipsilateral sinusitis, facial pain, fetor, and headache.^5^–^7^,^9^ In longstanding cases without medical intervention, dacryocystitis and otorrhea have been described. Severe cases can result in local destruction resulting in septal perforations as well as oroantral fistula.^5^,^11^,^13^,^14^

A common presenting history of exogenous stone formation is of a foreign body placed in childhood that was never removed. Less common is iatrogenic rhinolithiasis from retained nasal packing, sutures, or gauze that facilitate stone formation. Rare reports include concreted topical ointments, debris from nasopharyngeal refluxed emesis, and even stagnant inhaled particulates in the setting of impaired nasociliary clearance.^10^ Stones of endogenous origin are very rare, but osseous fragments following facial trauma, inspissated secretions, microemboli, and ectopic teeth have all been described.^12^

The vast majority of rhinoliths are observed along the floor of the nasal passage abutting the inferior turbinate; however, they have been described throughout the sinonasal passage.^11^,^13^,^15^ Removal is curative with minimal side effects in most cases with rare reports of postoperative bleeding and infection.^9^

In this case, an adult male presented to the ED with unilateral nasal obstruction and epistaxis. Computed tomography was appropriately ordered to further classify the mass, and subspecialty referral to otolaryngology was made. Treatment involved surgical removal with no need for antibiotics. Notably, the core of the calcified mass was seen to have a firm, rubbery texture with illegible orange foreign text. Given his history of endoscopic sinus surgery while living in Afghanistan, it is likely this substance represented a retained Penrose drain, which is occasionally used in sinus operations. Rhinolith is an uncommon clinical etiology; however, nasal obstruction and epistaxis are commonly encountered in acute care clinics. Given the broad differential for unilateral obstructing nasal masses, biopsy should be approached with caution.

## CONCLUSION

Rhinolithiasis is an uncommon etiology of unilateral nasal symptoms such as obstruction, pain, epistaxis, or purulent drainage. This is a slowly developing condition that is easily overlooked due to similarities in presentation with nasal neoplasms. If left unresolved, there is risk of local destructive change. Work-up of unilateral nasal mass, epistaxis, or purulent drainage without clear etiology should include CT, as biopsy is risky given the broad differential of unilateral mass. Surgical removal has a high success rate with low risk of complication.
